# QuickStats

**Published:** 2013-02-22

**Authors:** Tainya C. Clarke, Debra Blackwell

**Figure:**
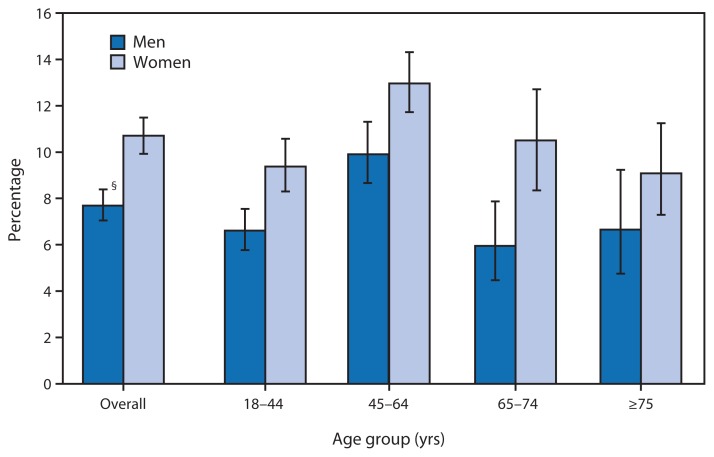
Percentage of Adults Aged ≥18 Years Who Often Felt Depressed,* by Sex and Age Group — National Health Interview Survey, United States, 2010–2011^†^ ^*^ Respondents were asked: “How often do you feel depressed? Would you say daily, weekly, monthly, a few times a year, or never?” Persons having daily or weekly feelings of depression were categorized as often depressed. Unknowns were not included in the denominators when calculating percentages. ^†^ Estimates are based on household interviews of a sample of the U.S. civilian, noninstitutionalized population. ^§^ 95% confidence interval.

During 2010–2011, women were more likely than men to often feel depressed (10.7% compared with 7.7%), overall and among those aged 18–44, 45–64, and 65–74 years. For both men (9.9%) and women (13.0%), the prevalence of depression was highest among those aged 45–64 years.

**Source:** National Health Interview Survey, 2010 Quality of Life and 2011 Functioning and Disability supplements. Data are from a subset of the adults randomly selected for the Sample Adult Component of the National Health Interview Survey questionnaire. Available at http://www.cdc.gov/nchs/nhis.htm.

